# Soil moisture gradients strengthen mesoscale convective systems by increasing wind shear

**DOI:** 10.1038/s41561-025-01666-8

**Published:** 2025-04-04

**Authors:** Emma J. Barton, Cornelia Klein, Christopher M. Taylor, John Marsham, Douglas J. Parker, Ben Maybee, Zhe Feng, L. Ruby Leung

**Affiliations:** 1 UK Centre for Ecology and Hydrology, Wallingford, UK; 2 National Centre for Earth Observation, Wallingford, UK; 3 School of Earth and Environment, University of Leeds, Leeds, UK; 4 National Centre for Atmospheric Science, Leeds, UK; 5 NORCE Norwegian Research Centre AS, Bergen, Norway; 6 Atmospheric, Climate, and Earth Sciences, Pacific Northwest National Laboratory, Richland, WA USA

**Keywords:** Atmospheric dynamics, Natural hazards

## Abstract

Mesoscale convective systems are a class of storm linked to extensive flooding and other destructive hazards in many regions globally. In West Africa, soil moisture impacts provide a valuable source of predictability for mature storm hazards, but little is known about mature storm sensitivity to soil moisture in other climatic regions. Here we use a storm track dataset, satellite observations and reanalysis fields to investigate the response of mature storms to soil moisture in seven global storm hotspots—West Africa, India, South America, South Africa, Australia and the United States Great Plains. We demonstrate that mesoscale soil moisture gradients (~500 km) can enhance storms by driving increased vertical wind shear conditions, a crucial ingredient for storm organization, through the strengthening of atmospheric temperature gradients. This is evidenced by a 10–30% increase in precipitation feature size and rainfall for the largest storms on days with favourable soil moisture gradients compared with unfavourable gradients. Global simulations confirm that soil moisture gradients influence wind shear. The results demonstrate the importance of soil moisture feedbacks for accurate forecasting of mesoscale convective systems and future projections of extreme events under climate change.

## Abstract (web summary)

Large-scale soil moisture gradients can lead to increases in vertical wind shear that promote the growth of mesoscale convective systems and enhance rainfall, according to an analysis of global hotspots of these extreme storm systems.

## Main

In many regions of the world, organized thunderstorm clusters known as mesoscale convective systems (MCSs) are the primary cause of extreme weather, such as destructive winds, lightning, flash flooding and hail. Representing some of the most intense thunderstorms on this planet^1^, they are strongly favoured in ‘hotspot’ regions such as West and Central Africa, Argentina, Northern India, China and the United States Great Plains, where their contribution to rainfall statistics is substantial. Over tropical land, they produce 50–90% of total rainfall^2^, with long-lived MCSs contributing the vast majority of extreme rainfall days^3^.

The relative importance of different atmospheric drivers for MCS organization and intensity varies across regions and seasons. Ingredients known to favour MCS development include high convective available potential energy, atmospheric moisture convergence and wind shear^4,5^. Idealized model studies suggest that wind shear can enhance cloud–cloud interactions^6^, modify the MCS-relative inflow of moist unstable air^7^, and reduce entrainment^8^, thereby enhancing MCS upscale growth and rainfall intensity^9–11^. Around the world, the most intense MCSs develop in baroclinic zones under sheared conditions^12–14^, conditions that may strengthen under global warming. Indeed, increased baroclinicity has already been linked to a substantial intensification of Sahelian MCSs in recent decades^15^.

Land surface processes have been shown to influence these atmospheric MCS drivers^16^. However, such effects barely feature in the literature and are missing from recent MCS state-of-knowledge reviews^2^. Observational studies from around the world have illustrated how spatial variations in surface sensible heat flux (*H*) on scales of <50 km favour the initiation of convection by driving daytime mesoscale circulations^17–21^. In the Great Plains, memory from springtime soil moisture (SM) was found to affect nocturnal MCS growth in the summer^22^. In the Sahel, dry patches on scales >100 km were found to increase the scale and longevity of propagating, mature afternoon MCSs via a combination of locally enhanced *H*, convergence and increased wind shear^23^. Such surface features can enhance the predictability of severe convection^24^.

**Fig. 1:**
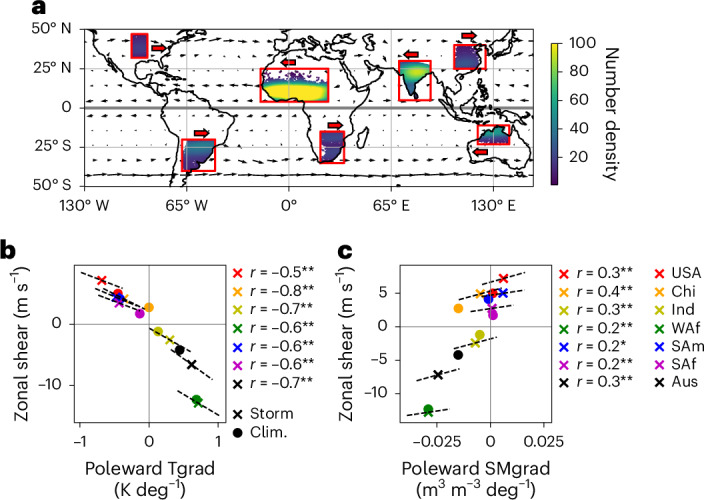
Global overview of mesoscale convective systems and pre-storm environment. **a**, Distribution of wet season MCS cases (colours) and season mean 650-hPa driving winds (black arrows). Wet season denotes May–September and November–March for the Northern and Southern Hemispheres, respectively. Red boxes highlight the study regions. Red arrows indicate the dominant zonal storm propagation direction. **b**,**c**, Scatter plots of regional mean pre-MCS conditions. Crosses represent MCS day conditions; circles denote climatology (clim.). Dashed gradient lines represent individual region linear regressions with correlation coefficients provided in the legends (* and ** indicate significance at the 95% and 99% levels, respectively). **b**, Poleward 925-hPa atmospheric Tgrad versus zonal shear. **c**, Poleward SMgrad versus zonal shear. Zonal shear is calculated between levels 650 hPa and 100 m. How the observed (ERA5) zonal shear relates to theoretical thermal wind is presented in Supplementary Fig. 6.

Synoptic- to continental-scale *H* gradients can modulate atmospheric temperature gradients and the associated vertical wind shear^25,26^, potentially feeding back on MCS characteristics^27^. In this Article we examine whether SM gradients affect variations in shear and the properties of mature MCSs across multiple storm hotspot regions: West Africa (WAf), India (Ind), South Africa (SAf), South America (SAm), Australia (Aus), China (Chi) and the Great Plains (USA) (Fig. 1a). We exclude forested MCS hotspot regions where surface fluxes are less sensitive to SM.

Based principally on satellite observations of MCSs and antecedent land conditions, we apply a consistent approach to the different regions using global datasets. Using a satellite-based high-resolution MCS track database^28^, we identify afternoon to early-evening MCSs that initiate 1–7 h before sampling. We characterize the land surface state using pre-MCS satellite observations of SM and interpret mechanisms linking surface states with MCS properties using ERA5 reanalysis. We also exploit global SM sensitivity simulations from the Coupled Model Intercomparison Project Phase 6 (CMIP6) LS3MIP experiment^29^ to confirm shear sensitivity to SM.

## Surface and atmospheric conditions preceding MCSs

First we examine morning surface and atmospheric conditions preceding afternoon MCSs. We focus on the wet season (May–September or November–February for the Northern or Southern Hemisphere, respectively) when the majority of MCSs occur (Fig. 1a). We consider only MCSs that initiate after 12:00 local time (LT) to ensure morning observations represent pre-MCS conditions. We consider time steps when a tracked MCS exceeds 2,500 km^2^ in area, and focus our analysis on the largest contiguous precipitation feature (PF1) within the MCS. We identify PF1 locations between 14:00 and 20:00 LT, retaining only features with maximum rain rates of >8 mm h^−1^.

We compute spatial composites centred on PF1 locations of pre-MCS SM from the Soil Moisture Active Passive satellite (SMAP, 6:00 LT) and ERA5 925-hPa temperature (10:00 LT) (Supplementary Figs. 2–4), from which we sample poleward gradients (Methods). We find significant (*P* < 0.05, −0.7 ≤ *r* ≤ −0.2) correlations between gradients in SM (SMgrad) and temperature (Tgrad; Supplementary Fig. 5).

Horizontal Tgrad is expected to influence vertical wind shear, a key ingredient for MCS growth, through the thermal wind relation. As the observed composite mean gradients are predominately meridional in orientation (Supplementary Figs. 2–4), we sample pre-MCS low-level zonal shear (650 hPa–100 m; Methods), the dominant direction of which also corresponds to the main MCS propagation directions in the respective regions (Fig. 1a). We observe significant correlations (0.5 ≤ *r* ≤ 0.8, *P* < 0.001) between poleward Tgrad and zonal shear (Fig. 1b) in all regions, consistent with shear strengths derived from the thermal wind relation (Supplementary Fig. 6). Poleward SMgrad and shear are similarly significantly correlated (*P* < 0.05; Fig. 1c), although the relationship is weaker (0.2 ≤ *r* ≤ 0.4). Hence, although SMgrad explains 4–50% (*r*^2^) of Tgrad variability, this reduces to 4–15% of shear variability depending on the region. Causality between SMgrad and shear is addressed later in ‘Shear sensitivity to soil moisture globally’. Note in Fig. 1b,c that gradients and shear conditions on MCS days are systematically enhanced for most regions compared to their climatology, suggesting that days favourable for MCS development tend to feature higher shear, consistent with regional studies^4,30,31^.

## Influence of shear environment on MCS characteristics

**Fig. 2:**
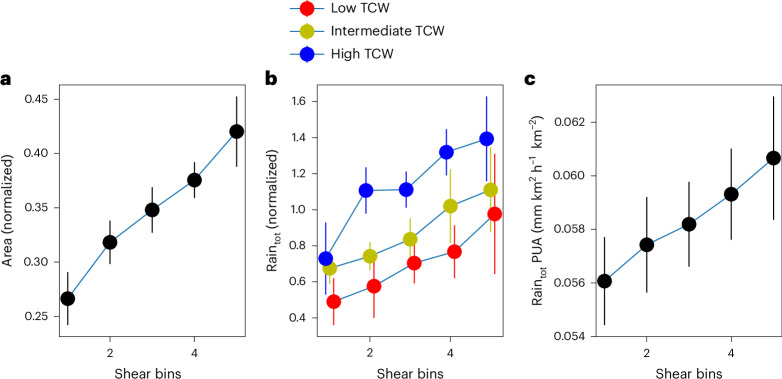
Impact of zonal shear environment on wet season MCS characteristics (largest precipitation feature, PF1) across all regions. **a**, Line plot of mean normalized PF1 area in regular zonal shear bins (low to high). **b**, Line plots of 95th percentile normalized PF1 rain_tot_ in regular zonal shear bins (low to high) for low (red), intermediate (yellow) and high (blue) TCW. **c**, Line plot of mean rain_tot_ per unit area (PUA) (mm km^2^ h^−1^ per km^2^) in regular zonal shear bins (low to high). Data in **a**–**c** are presented as mean values ± 95th percentile confidence intervals (*n* = 7 regions). Variables in **a**,**b** are normalized for each region by expressing the value as a fraction of the largest value for that region (Methods). Absolute values for each region (including shear ranges and TCW subsets) are presented in Supplementary Fig. 7. Data points in **b** have been plotted with an *x*-axis offset to aid visualization of error bars. Zonal shear is calculated between levels 650 hPa and 100 m.

To analyse the impact of zonal shear on precipitation feature characteristics, we consider PF1 size and total hourly rainfall (rain_tot_) in equally spaced shear (and total column water (TCW) for rain) bins. Pooling all regions together (Fig. 2a), there is a strong relationship between shear and PF1 size (Methods), with a 60% increase (~13,000 km^2^; Supplementary Fig. 7) in PF1 area across the shear range (~10 m s^−1^). Shear influences internal circulations within a MCS and can thereby promote organization and growth^32,33^. TCW is an important control on rain_tot_, as evidenced in Fig. 2b by increases of up to 50% in rain_tot_ between low and high TCW. Rain_tot_ also increases with shear by 100%, 80% and 130% on average across the shear range for low, moderate and high TCW, respectively. The increase in PF1 rain_tot_ may not solely be due to scale growth, as suggested by a significant (*P* < 0.05) ~10% increase in its area-normalized value (Fig. 2c). This motivates further investigation into dynamical feedbacks, such as increased moisture inflow^34^ and/or decreased entrainment^8^. Note that qualitatively similar (though noisier) relationships between PF1 characteristics and shear are found when considering regions separately (Supplementary Fig. 7), particularly for PF1 size. We observe an ~6,000-km^2^ (SAf) to 25,000-km^2^ (USA) increase in PF1 area, and a 500-mm (Aus high TCW) to 8,000-mm (USA low TCW) increase in rain_tot_.

## Impact of SM gradient on MCS characteristics

We now consider how variability in pre-storm poleward SMgrad impacts MCS characteristics. We create two subsets of the MCS data based on whether the anomalous SM gradient (SMAgrad) provides favourable or unfavourable contributions to wind shear in the direction of MCS propagation. The subsets contain cases within the upper and lower quartiles of the poleward SMAgrad distribution. We use anomalies here to focus on MCS day conditions and limit the analysis to cases with sufficient SMAP data to sample SMAgrad (≥100 cases per quartile; Methods). This condition limits our results to WAf, Ind, SAf and SAm.

**Fig. 3:**
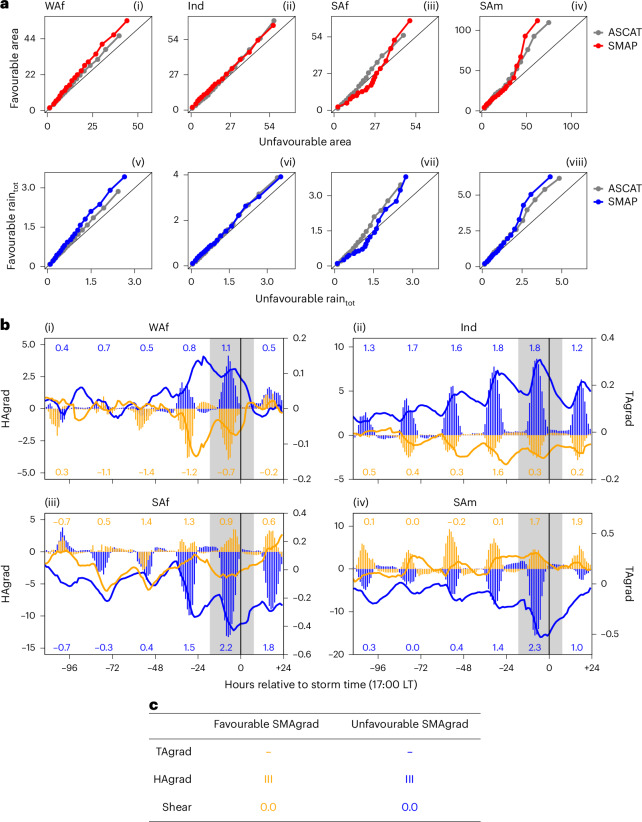
Differences between the wet-season MCS characteristic (PF1) distributions and temporal evolution of atmospheric and surface variables for favourable and unfavourable soil moisture gradient anomalies in four regions. **a**, Quantile–quantile (Q–Q) plots comparing 5th to 95th (in steps of 5) percentile values of PF1 area × 10^3^ (km^2^) (i–iv) and total rainfall (m km^2^ h^−1^) (rain_tot_, v–viii) for favourable and unfavourable SMAgrad subsets defined using SMAP (coloured) or ASCAT (grey). Differences in the favourable and unfavourable distributions are indicated by deviation from the 1:1 line (black). Absolute and percentage differences in the percentile values for SMAP are shown in Supplementary Fig. 8. Favourable refers to SMAgrad that enhances shear in the direction of MCS propagation. **b**, Subset mean diurnal cycles of poleward sensible HAgrad W m^−2^, bars) and poleward atmospheric TAgrad anomalies (K) for favourable (blue) and unfavourable (orange) SMAgrad subsets (SMAP). Annotations quantify the subset mean daily average 650 hPa–100 m zonal shear anomaly (shearA) (m s^−1^), where positive values indicate an enhancement of shear in the direction of MCS propagation. Grey shading highlights storm day. **c**, Legend for **b**. Hgrad anomalies (vertical bars), Tgrad anomalies (soild lines) and shear anomalies (number annotations) for the favourable and unfavourable SMAgrad subsets (SMAP) are represented by blue and orange colours, respectively. The spatial and temporal distributions of cases in the favourable and unfavourable SMAgrad subsets for each region are presented in Supplementary Fig. 13.

Figure 3a compares the distributions of PF1 area (i–iv) and rain_tot_ (v–viii) for the favourable and unfavourable subsets per region. These show that the favourable distributions are shifted towards larger PF1s that produce more rainfall, with a 10–50% increase in PF1 size and rain_tot_ for the 90th percentile (SMAP; Supplementary Fig. 8). Qualitatively similar results are found using an alternative SM dataset (ASCAT), yielding an increase of 15–40% in PF1 size and rain_tot_ (Fig. 3a and Supplementary Fig. 9). This range is 12–30% when controlling for TCW (Supplementary Figs. 10–12), suggesting a partial contribution from TCW variability. Although the choice of SM dataset introduces uncertainty in the magnitude of enhancement, overall this indicates that favourable antecedent SM conditions coincide with larger, more precipitating MCSs.

Using ERA5 data, Fig. 3b illustrates the contrasting evolution of lower-atmospheric Tgrad between the two subsets per region in the days ahead of PF1. To ensure diurnal cycle consistency across cases, we focus on PF1 locations sampled at 17:00 LT. Growth (in absolute terms) in Tgrad anomalies (TAgrad) preceding the MCS only occurs during the daytime. This demonstrates that the differential heating with latitude originates from diurnally varying surface fluxes. Unmediated by the land surface, synoptic processes could not create this phase-locking of low-level heating to the diurnal cycle. The role of surface fluxes is confirmed by the time series of anomalous *H* gradients (HAgrad) in ERA5, which increase in amplitude over the days preceding the MCS, with the expected diurnal cycle superimposed. Given favourable SMAgrad, TAgrad in each region strengthens over this period, and is accompanied by increasingly favourable shear.

The number of days over which strong differential warming develops varies across regions. In Ind and SAm, favourable heat flux, temperature and shear conditions develop gradually over four days (Fig. 3b(ii) and (iv)), whereas in WAf/SAf, clear differences between favourable and unfavourable subsets only emerge in the two days before the MCS (Fig. 3b(i) and (iii)). This probably reflects regional differences in characteristic rainfall frequencies and evaporation sensitivities to dry spells^35^. In all regions, HAgrad and TAgrad are weakened after the MCS, presumably affected by rainfall from the MCS itself (Fig. 3b).

To test whether detected favourable anomalies are consistent with theory, we estimate the expected magnitude of anomalies in Tgrad and shear given HAgrad and TAgrad, respectively (Methods). Assuming a 2-km deep boundary layer, the range of accumulated MCS-day HAgrad across regions would increase Tgrad by ~0.1–0.2 K, consistent with the TAgrad increase on MCS day (Fig. 3b). Using the linear regressions from Fig. 1, pre-MCS values of TAgrad would enhance shear by ~1–4 m s^−1^, consistent with the MCS day shear anomaly (Fig. 3b). Taken together, this provides quantitative evidence that the detected variability in SMgrad is strong enough to influence wind shear via Tgrad.

## Shear sensitivity to soil moisture globally

**Fig. 4:**
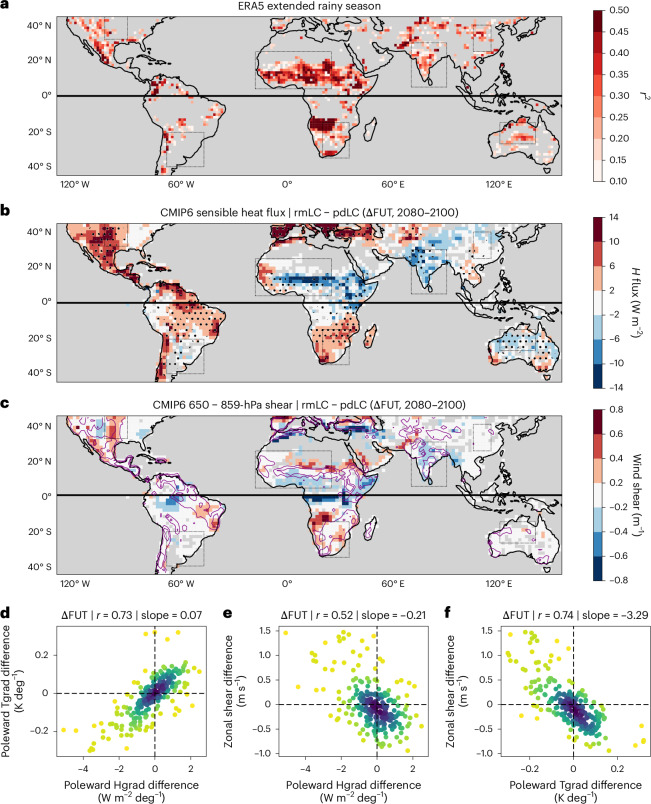
Shear sensitivity to SM gradients (SMgrad) globally. **a**, Maximum *r*^2^ for inter-annual monthly correlations (*r*) between meridional SMgrad and zonal wind shear (ERA5, 1980–2020). Only pixels with significant (*P* < 0.05) shear correlation with SMgrad and Hgrad are shown. **b**,**c**, Average future differences (ΔFUT, 2080–2100) for five CMIP6 LS3MIP models between experiments using prescribed future climatological SM (rmLC) and prescribed historical climatological SM (pdLC) for the resulting *H* flux (W m^−2^) (**b**) and wind shear (m s^−1^) (**c**). Oceans and pixels where no model shows agreement between signs of *H* flux and temperature changes are grey. Stippling in **b** indicates pixels where *H* flux and temperature changes agree in sign for all CMIP6 models, that is, where flux and temperature changes are consistent. Purple contours show absolute *H* change (4–8 W m^−2^ lines) from **b**, for comparison. **d**–**f**, Scatter plots illustrating the ΔFUT relationships for Hgrad differences (W m^−2^ deg^−1^) with the co-located poleward Tgrad differences (**d**), Hgrad (W m^−2^ deg^−1^) differences versus zonal wind shear (m s^−1^) differences (**e**) and Tgrad differences versus zonal wind shear differences versus Hgrad (W m^−2^ deg^−1^) (**e**) and Tgrad differences versus zonal wind shear differences (**f**) in MCS hotspot regions (dotted rectangles in **a**–**c**) for the five CMIP6 models. Only land pixels where signs of *H* and temperature changes agree are included. The data in **a**–**f** were calculated within extended rainy seasons for the respective hemisphere (North/South split).

Next we use model data to identify regions where wind shear significantly co-varies with changes in Hgrad, and therefore where MCSs may be sensitive to changes in SMgrad. In Fig. 4a, we consider maximum inter-annual correlations between monthly ERA5 meridional SMgrad and zonal shear (Methods). Areas with high correlation between SMgrad and shear include the monsoon regions in Asia, Africa and Australia, all of which exhibit strong aridity gradients and MCS development. These are also strong land–atmosphere coupling regions^36^. Notably, however, spatially limited shear correlation with SMgrad is evident in the USA and SAm, where frequent summertime MCS development is well known, but shear is dominated by meridional low-level jet variation rather than zonal wind responses^25,37^.

Finally, to explore the direct effect of SM changes on total (meridional and zonal) shear, we use LS3MIP experiments available for five CMIP6 models^29^, with one experiment using prescribed future SM climatology (rmLC), and a second using prescribed historical SM climatology through to year 2100 (pmLC). LS3MIP thus allows a clean, multi-model evaluation of SM effects on shear, where the forcing corresponds to projected SM changes in CMIP6 SSP5-8.5 simulations (2080–2100, rmLC minus pdLC). This means that any difference in Hgrad is by definition induced by differences in imposed SMgrad between the two simulations, with expected effects on wind shear. Regions with locally strong SM-driven changes in *H* (and therefore changes in Hgrad) in Fig. 4b tend to exhibit adjacent patterns of changes in total wind shear in Fig. 4c. Examples include patterns along the northern Sahel, over Pakistan and in response to a strengthened zonal flux gradient in the region of the United States Great Plains low-level jet. Regions that currently experience sheared conditions but where SM gradients are not projected to change markedly, accordingly exhibit no or very weak shear differences, as is evident in Chi and Aus. Not all the MCS hotspot regions defined in Fig. 1 show a marked change in surface gradients in the simulations (Fig. 4b), so areas of shear sensitivity are not expected to be consistent with these regions.

Evaluating zonal shear differences as a function of poleward Tgrad differences across all MCS hotspot domains reveals a clear relationship (Fig. 4d) that is consistent with the shear response to poleward Hgrad differences illustrated in Fig. 4e, where regions with small Hgrad differences similarly exhibit weak Tgrad (Fig. 4f) and shear differences across the SM-prescribed CMIP6 simulations. These direct SM-driven effects on shear via Hgrad (Fig. 4e,f) illustrate the importance of SM for the modification and positioning of sheared environments that create favourable conditions for convective organization.

## Discussion

We have shown that anomalous SM gradients on scales of several hundred kilometres are strong enough to enhance shear and thereby favour larger and more heavily precipitating MCSs. This pathway for SM to reinforce storms is relevant across seven global MCS hotspot regions that collectively are home to several billions of people. The links within the pathway are individually well established in the literature^9–11,15^. We demonstrate that a signal of day-to-day SM variability translates into 10–30% increases in precipitation area for the largest MCSs, even when co-variability in thermodynamic drivers and dataset differences is considered. We thus present a consistent process chain-linking SM gradients to MCS properties across all studied regions. However, the strength of the regional relationships is expected to differ for several reasons, including the sensitivity of surface fluxes to SM, the relative importance of shear versus moisture variability for MCS characteristics, and the latitude dependence of the thermal wind.

We note that an observation-based demonstration of the impact of the strongest SMgrad on MCS characteristics is only possible for four of our hotspot regions, as the SMAP dataset length limits subsetting. However, we have shown correlations between surface/atmospheric gradients and shear, and MCS sensitivity to shear for all our regions based on the global datasets. Overall, this study probably presents an underestimate for the control of SM on Tgrad, as the atmospheric state within reanalysis data is coupled to an imperfect representation of the observed SMgrad. Similarly, SMgrad and shear relationships have not been filtered for synoptic conditions, so will represent an underestimate for days with weak synoptic forcing.

Our results indicate MCS intensification by favourable SMgrad that persists over two to five days, suggesting predictive potential. Frequent assimilation of satellite-derived SM within the emerging generation of global convection-permitting models thus offers the prospect of improved forecasting of hazardous weather. This is of particular importance in data-sparse and climatically exposed regions such as Africa, where 60% of the population currently lack early warning systems^38^. Future research should consider controlled SM experiments at convection-permitting scales to further explore the region-specific MCS sensitivity to the described process chain. Concurrently, even fine-scale convection-permitting simulations can fail to capture shear effects^39^, highlighting the need to scrutinize MCS sensitivities in our models. On climate-change timescales, MCSs are projected to become more intense but less frequent in regions with strong aridity gradients^40,41^, a combination that is expected to intensify mesoscale SMgrad. Our findings indicate that stronger SMgrad will result in stronger shear and hence larger and more precipitating MCSs, so our observed feedback could strengthen under climate change.

## Methods

Throughout this work, we use the near-global (60° S–60° N), long-term (2000–2019) MCS dataset version 1 developed by Feng and colleagues^28^. At a resolution ~10 km, the dataset contains details of MCSs tracked jointly using geostationary satellite infrared brightness temperature (*T*_b_) and precipitation feature (PF) characteristics from the Integrated Multi-satellitE Retrievals for GPM precipitation datasets. MCSs were defined as cloud systems with a maximum cold cloud shield (*T*_b_ < 241 K) area greater than 40,000 km^2^ that persisted longer than 4 h, with additional constraints on the size and intensity of PF characteristics. Here we only consider stages in the storm life cycle when the MCS has PFs larger than 2,500 km^2^ with a maximum rain rate of >8 mm h^−1^. This excludes weaker storm stages and periods dominated by stratiform rain^42^. We focus on afternoon and early-evening MCSs, retaining only those systems that initiated after 12:00 LT and existed for at least 1 h. Note that we consider all MCSs that comply with these conditions, regardless of distance travelled from initiation location. Within these MCSs, we consider the largest precipitation feature (PF1), as identified by the tracking algorithm. We note that the thresholds used to define MCSs in this global MCS tracking dataset favour larger systems, so the number of cases in regions with a greater proportion of smaller systems is limited (for example, Chi), and MCS numbers may diverge from region-specific datasets that use adapted thresholds and thus include smaller, shorter-lived MCSs.

We analyse a combination of satellite-derived SM observations and atmospheric reanalyses to characterize the environment of the coupled land–atmosphere system in which these MCSs develop. We sample both background conditions and anomalies (background minus ± 15-day climatology). We sample background conditions to understand how MCSs are responding to the environment to which they are exposed. We sample anomalies to understand the impact of storm day-specific conditions. For SM, we use observational datasets produced by both the SMAP^43^ mission and ASCAT^44^. These were chosen as they have morning overpasses (around 6:00 and 9:30 LT, respectively) before daytime convection tends to develop. They have been providing data since 2015 (SMAP) and 2007 (ASCAT). We use SMAP as our primary dataset due to the earlier overpass time and higher resolution (9 km versus 25 km). For other variables we use the ERA5^45,46^ reanalysis dataset, which provides hourly data at the surface and on a range of single and pressure levels with a spatial resolution of 0.25°.

### Atmospheric and surface composites and gradients

For the composite data in Fig. 1 and Supplementary Figs. 2–6, we consider only the years 2015–2019, when SMAP data are available. We extract the location of PF1 from the MCS database between 14:00 and 20:00 LT (or for as many hours as the maximum rainfall rate exceeds 8 mm h^−1^). We then sample the pre-MCS environment for each PF1 location. Where an MCS persists and produces rainfall in excess of 8 mm h^−1^ for multiple time steps within this window, it contributes additional PF1 locations (at different times) to this sample. Supplementary Table 1 provides case numbers and Supplementary Table 2 the track statistics. Pre-MCS atmospheric conditions from ERA5 are sampled at 10:00 LT. As parameterized convection in ERA5 has a tendency to develop earlier than in observations, we exclude cases where vertical velocities at 500 hPa exceed 0.4 ms^−1^ within 100 km of PF1 at 10:00 LT. We also exclude cases where rainfall in ERA5 exceeds 1 mm within 100 km of PF1 between 6:00–10:00 LT.

To produce the spatial composites we sample a 6 × 6° box from the data (satellite or reanalysis) centred on PF1. We then take a mean of this for all cases. We do not remove seasonal and diurnal cycles before compositing, as we are interested in the background conditions. For the satellite-based composites we exclude cases with less than 25% valid pixels in the domain. For the ERA5 composites we mask pixels over the ocean to ensure we are focusing on the land. To compute gradients we again sample a 6 × 6° box from the data (satellite or reanalysis) centred on PF1. For each case we take a zonal mean of the values in the domain, and sample the gradient between ±3° N/S. For gradients derived from satellite data we exclude cases that have less than three pixels contributing to the zonal mean at ±3° N/S. For shear conditions we compute the difference in zonal winds between 650 hPa and 100 m, taking a domain mean in the 6 × 6° box, excluding pixels over the ocean. These levels were selected to sample mid- and low-level flow at consistent levels in all regions. We opted for 100 m as the lower level (rather than a pressure level) to ensure that the variables were being sampled above the surface, even for higher-altitude locations (for example, southern Africa).

### Sensitivity of MCSs to shear

To analyse the sensitivity of PF1 characteristics (area, total hourly rainfall and total hourly rainfall per unit area) to shear, and TCW for total rainfall, we consider the full time coverage of the track dataset (2000–2019). Case numbers are recorded in Supplementary Table 1.

The impact of shear on MCSs is dependent on direction. To reduce complexity, PF1 cases are filtered for the dominant MCS propagation direction in each region (westwards or eastwards). Westwards (eastwards) is defined as a heading between 225 and 315° (45 and 135°). To make the relationships easily comparable, we define shear in the direction of MCS propagation as positive. We first produce the relationships between shear and MCS characteristics for each region separately. For the sensitivity of the PF1 area to shear (Fig. 2a), cases are subset by shear conditions into five evenly spaced shear bins for each region, with bin widths ranging from 1.8 to 2.7 m s^−1^. We then compute the mean PF1 area within each shear bin. For the cross-region average (Fig. 2a), the PF1 area for each region is normalized as a fraction of the largest PF1 area for that region. This is to reduce the impact of regional differences in MCS size on the cross-region mean relationship. We then take an average of the normalized regional means for each shear bin. We adopt a similar approach for the sensitivity of total rainfall to shear (Fig. 2b), but additionally subset the cases in each region into three equally spaced TCW groups. Each TCW group is then subset by shear conditions into five evenly spaced shear bins. For each TCW region group, we then compute the 95th percentile total rainfall within each shear bin. For the cross-region average (Fig. 2b), each regional PF1 total rainfall is normalized by the highest PF1 total rainfall for that region, again to reduce the impact of regional differences in the cross-region mean relationship. Calculating the sensitivity of total rainfall per unit area to shear (Fig. 2c), we again create five bins for each region, based on total rainfall per PF1 divided by PF1 area for each case. The multi-region average (Fig. 2c) is then an average of the regional means for each shear bin.

### Demonstrating the impact of SM on MCS characteristics

To compare MCS characteristics between favourable and unfavourable SM conditions (Fig. 3), we first select MCSs with a PF1 at 17:00 LT (with maximum rainfall rate exceeding 8 mm h^−1^). These MCSs are subset into quartiles of SMA gradient (6:00 LT observations sampled at the 17:00 LT PF1 location), and the upper and lower quartiles are retained. Regions with fewer than 100 cases per quartile are excluded. We then extract the characteristics of PF1 for each MCS from its track between 17:00 and 0:00 LT or until the track ends (MCS dissipation). We further exclude time steps where the MCS has travelled further than 2° from its position at 17:00 LT (and may therefore be exposed to different surface conditions). MCS numbers for each region are recorded in Supplementary Table 1.

To investigate our proposed mechanism linking SM to shear (and therefore MCS characteristics) for the two SMA quartile MCS subsets, we sample the hourly evolution in ERA5 of north–south gradients in the surface sensible heat flux anomaly and the 925-hPa temperature anomaly at the 17:00 LT PF1 location for the period starting 120 h before the event to 24 h after. We also sample daily mean shear anomalies. As before, we sample a 6 × 6° box from the data (ERA5) centred on PF1, taking a domain mean for the shear or, for the gradients, an east–west mean of values then the north–south gradient ±3° from the centre. Hourly anomalies are calculated with respect to a ±15-day 20-year (2000–2019) climatology.

To test whether the observed temperature gradient and shear anomalies are consistent with theory, we perform simple calculations to convert (1) observed (ERA5) sensible heat gradient anomalies to temperature gradients anomalies (equation (1)), then (2) observed (ERA5) temperature gradient anomalies to shear anomalies (linear regressions from Fig. 1) for comparison to the observed (ERA5) values.

The theoretical increase in temperature in the planetary boundary layer is calculated using the formula for isobaric warming:

1 $${\Delta T}={\frac{\Delta Q}{{C}_{\rm{p}}M}}$$

where *M* is the mass of air (in kg), *C*_p_ is the specific heat capacity of air (1,004 J K^−1^ kg^−1^) and Δ*Q* is the heat input (in J m^−2^). We consider a closed one-square-metre column of air with a density of 1.2 kg m^−3^ and a boundary layer height of 2 km.

The accumulated heat input from sunrise to 17:00 LT on a storm day (WAf/Ind/SAf/SAm = 34/66/106/82 W m^−2^; Fig. 3) would increase the air temperature in the column by ~0.1–0.2 K (WAf/Ind/SAf/SAm = 0.05/0.1/0.16/0.12 K).

Using the linear regressions from Fig. 1, the temperature gradient anomaly at storm time (WAf/Ind/SAf/SAm = 0.1/0.3/−0.5/−0.6 K; Fig. 3) would enhance the shear in the direction of storm propagation by ~1–4 ms^−1^ (WAf/Ind/SAf/SAm = 2.5/1.3/3.7/4.3 m s^−1^).

### Identifying global regions where shear responds to SM

Monthly fields of ERA5 SM, sensible heat (*H*) flux, latent heat flux (*L*), 925-hPa temperature (*T*) and 650–925-hPa zonal vertical wind difference (shear) are used to explore inter-annual co-variability between SM gradients and shear globally. We first calculate meridional SM, *H*, *L* and *T* gradients centred on each pixel ±3° via linear regression. The fields are then degraded to 1.5° grid resolution to calculate monthly inter-annual correlations (*r*) for the period 1980–2020 between zonal vertical wind shear and gradients for SM, *H*, *L* and *T*. Figure 4a then shows the maximum coefficient of determination (*r*^2^) between SM gradients and shear for each grid cell, considering all months within the respective extended wet season (May–September for the Northern Hemisphere and November–March for the Southern Hemisphere). However, we only plot pixels where shear correlation is significant for both SM and *H*, and where shear correlation coefficients exhibit the same sign for SM and *L*, and reverse sign for *H* and *T*. This ensures only pixels are shown where correlation direction is consistent with SM control on turbulent fluxes.

We also exploit the CMIP6 LS3MIP experiments to illustrate changes in vertical wind shear as a direct consequence of SM changes. The experiments include five models (MPI-ESM1-2-LR, CESM2, IPSL-CM6A-LR, EC-Earth3 and CNRM-CM6-1), which were run through to 2100 with prescribed 30-year running mean climatologies of SM fields (rmLC experiment), and again using only the 30-year historical SM field (pdLC experiment, fixed 1980–2014 SM). We use raw model outputs interpolated onto a common 1° grid. Here we concentrate on the resulting climatological *H* flux (Fig. 4b) and vertical wind shear differences (Fig. 4c) calculated from the rainy season average (May–September in the Northern Hemisphere and November to March in the Southern Hemisphere) for the future period 2080–2100 between the rmLC and pdLC experiments, which can be fully attributed to the differences in prescribed SM fields. Figure 4b,c presents the model average. For scatter plots that present zonal wind shear as a function of poleward 925-hPa Tgrad and Hgrad differences, we calculate gradients for individual models and every second grid point (2°) across ±3° via linear regression. These gradients are then matched with co-located 650–925-hPa zonal wind differences of the respective CMIP6 model. We only plot grid points that lie within our predefined MCS hotspot regions and for which the sign of the Tgrad and Hgrad change is the same, thereby focusing on grid cells where low-level temperature changes may co-vary with *H* flux changes.

## Online content

Any methods, additional references, Nature Portfolio reporting summaries, source data, extended data, supplementary information, acknowledgements, peer review information; details of author contributions and competing interests; and statements of data and code availability are available at 10.1038/s41561-025-01666-8.

## Supplementary information

Supplementary Information Supplementary Tables 1 and 2, Figs. 1–13 and Discussion.

## Data Availability

Unprocessed data are available from the following sources: the MCS Track Dataset (version 1), which is archived at the NERSC High Performance Storage System (HPSS):/home/f/feng045/GPM/; SMAP (10.5067/7KKNQ5UURM2W); ASCAT (https://data.eumetsat.int/data/map/EO:EUM:DAT:METOP:SOMO25); ERA5 (pressure levels) (10.24381/cds.bd0915c6); ERA5 (single levels) (10.24381/cds.adbb2d47); CMIP6 LS3MIP future LFMIP-pdLC (prescribed historical SM) versus future LFMIP-rmLC (prescribed 30-y running mean of SM) (https://aims2.llnl.gov/search).

## Code availability

Code and data arrays for the main figures are available from the GitHub repository at 10.5281/zenodo.14860081.
