# Supplementary material for: Soil moisture gradients strengthen mesoscale convective systems by increasing wind shear
Source: Nat Geosci. 2025 Apr 4;18(4):330–6. doi: 10.1038/s41561-025-01666-8 (PMC11981932; doi:10.1038/s41561-025-01666-8)
Supplement: Supplementary file 1 — Supplementary Tables 1 and 2, Figs. 1–13 and Discussion. [file 41561_2025_1666_MOESM1_ESM.pdf]

nature geoscience

Supplementary information

https://doi.org/10.1038/s41561-025-01666-8

Soil moisture gradients strengthen mesoscale convective systems by increasing wind shear

In the format provided by the authors and unedited

Soil moisture gradients strengthen mesoscale convective systems by increasing wind shear – Supplementary Information

Case Summary

Table S1 - Case Numbers

	PF1 Cases 2D Composites SMAP 2015-2019 (Figure S1)	PF1 Cases 2D Composites ASCAT 2015-2019 (Figure S2)	PF1 Cases 1D Gradients SMAP 2015-2019 (Figure 1)	PF1 Cases MCS Properties 2000-2019 (Figure 2)	Number of MCSs in SMAP quartiles lower/upper* 2015-2019 (Figure 3)	Number of MCSs in ASCAT quartiles lower/upper* 2015-2019 (Figure S9)
Great Plains (USA)	764	554	171	1752	N/A	N/A
China (Chi)	844	801	194	1880	N/A	N/A
India (Ind)	3548	3079	1740	5694	185 / 169	181/192
West Africa (WAf)	5894	5601	4068	22345	305 / 280	423/435
Australia (Aus)	1185	1360	349	2595	N/A	N/A
South Africa (SAf)	1679	1498	635	3049	104 / 111	136/134
South America (SAm)	1511	1440	540	3414	102 / 112	113/114

* representing the most negative and positive soil moisture gradient anomalies.

Table S2 - Track Statistics

Region	Total number of MCSs* (2000 - 2019)	Percentage of storms that form within the study region (of total MCSs 2000 - 2019)	Mean distance traveled between initiation and first PF1 sampling [km]
USA	717	97%	131
Chi	887	76%	231
Ind	3676	87%	158
WAf	6833	95%	120
Aus	1812	90%	147
SAf	2261	90%	150
SAm	1713	94%	148

* This number is smaller than PF1 cases as some MCSs contribute multiple PF1 locations

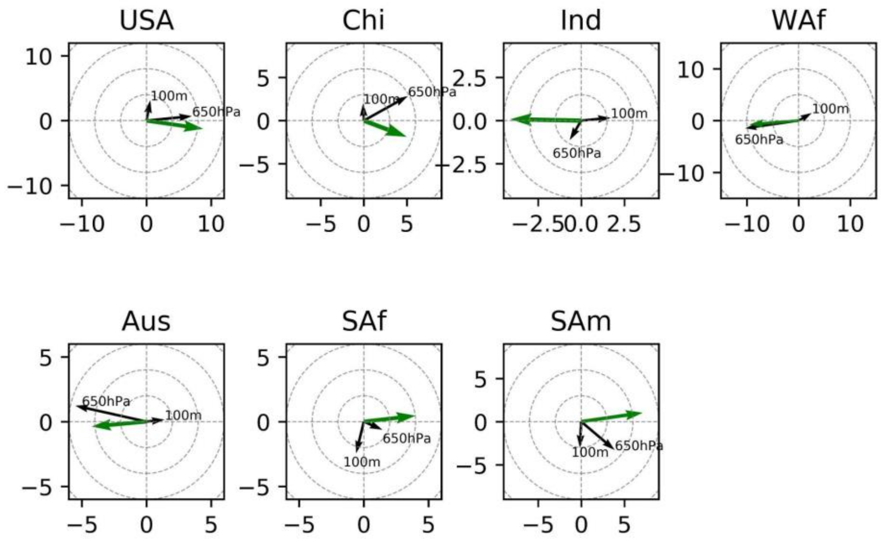

Figure S1 - Composite mean atmospheric conditions preceding MCSs. 1000LT hodographs, black arrows - domain mean 100 m and 650 hPa winds [ms^{-1}], bold green arrow - mean MCS propagation. Domain is a 6 x 6 degree box centered on PF1.

Surface and Atmospheric Conditions Preceding Storms

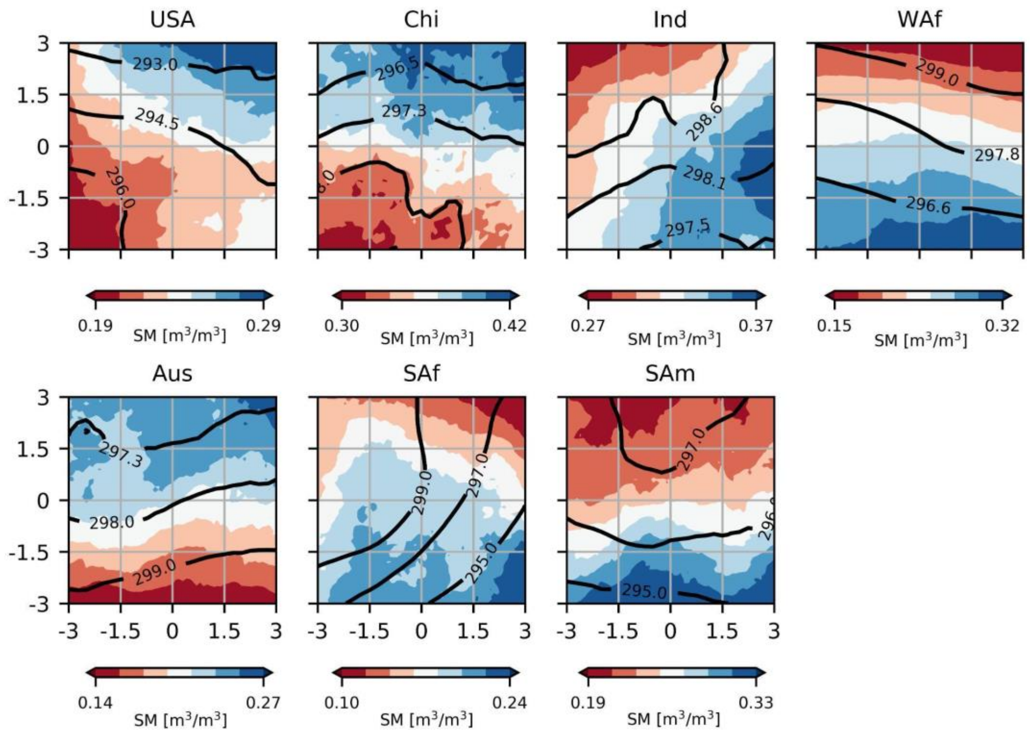

Figure S2 - Composite mean surface and atmospheric conditions preceding MCSs. Shading - SMAP 0600LT soil moisture (SM) [m^3/m^3], contours - ERA5 925 hPa air temperature at 1000LT [K].

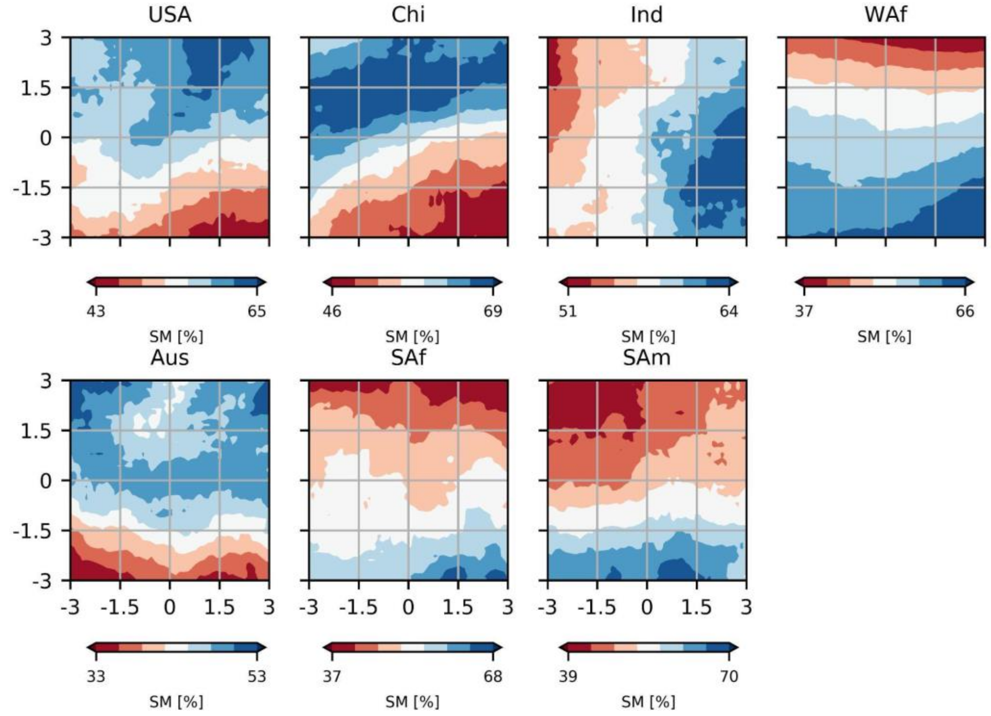

Figure S3 - Composite mean surface conditions preceding MCSs. Shading - ASCAT 1030LT soil moisture (SM) [saturation %].

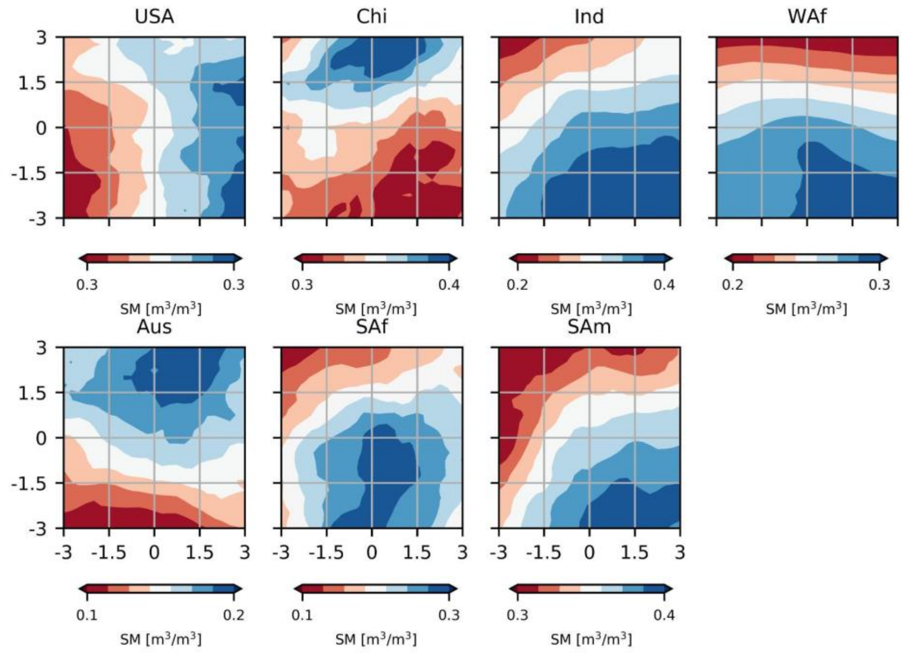

Figure S4 - Composite mean surface conditions preceding MCSs. Shading - ERA5 0600LT soil moisture (SM5) [m^3/m^3].

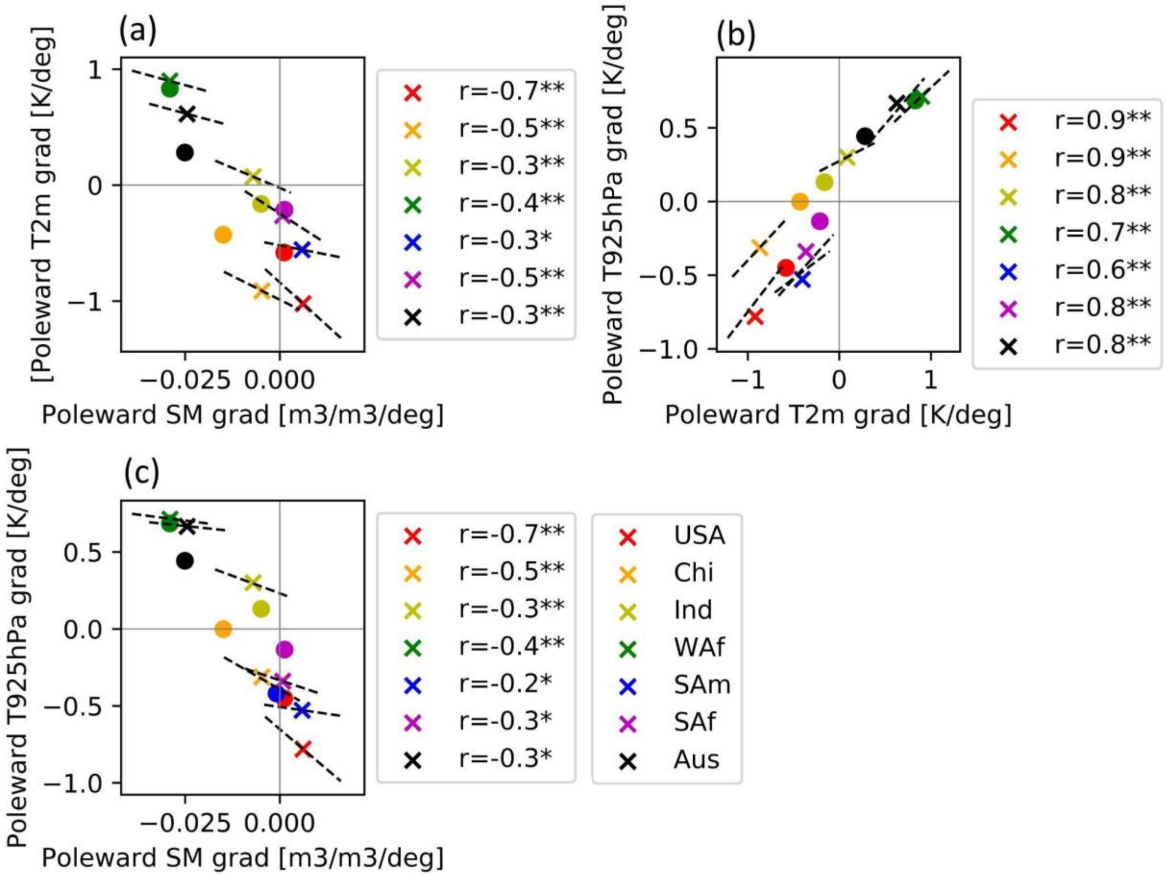

Figure S5 - Scatter plots of regional mean pre-MCS conditions. Crosses represent MCS day conditions, circles denote climatology. Dashed gradient lines represent individual region linear regressions with correlation coefficients provided in the legends, * / ** indicates significance at the 95 / 99% level. (a) Poleward soil moisture gradients (SMgrad) versus poleward 2m atmospheric temperature gradients (T2grad), (b) Poleward 2m atmospheric temperature gradients (T2grad) versus poleward 925hPa atmospheric temperature gradients (Tgrad) (c) SMgrad versus Tgrad.

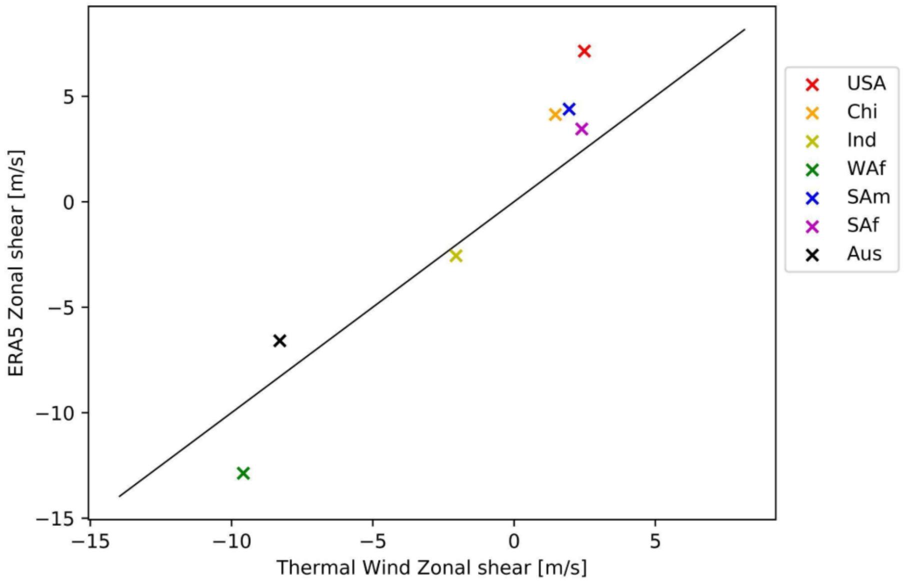

Figure S6 - Scatter plots of regional mean pre-MCS conditions. Observed (ERA5) zonal shear versus theoretical zonal shear ($p<0.05$). Observed (ERA5) zonal shear is calculated between levels 650h Pa and 100 m. Theoretical zonal shear is calculated using observed (ERA5) poleward 925 hPa atmospheric temperature gradients and the thermal wind relation (Equation S1).

Thermal Wind

Zonal thermal wind (shear) u_T ms^{-1} is calculated using the following expression for the thermal wind effect:

$$u_T \approx -\frac{g}{T_v f}\frac{\Delta T}{\Delta y}\Delta z \qquad (S1)$$

Where, g is the gravitational acceleration magnitude 9.8 ms^{-2}, T_v is the virtual temperature in K, f is Coriolis parameter s^{-1}, $\Delta T/\Delta y$ is meridional temperature gradient K/km (sampled from ERA5 at 925 hPa and assumed to be representative of the temperature gradient up to 650 hPa), Δz is the altitude change in km (assumed to be 3 km). T_v is taken to be the mean

temperature in the domain (sampled from ERA5 at 925 hPa), f is calculated from Equation S2 using the mean latitude for each region.

$$f = 2\Omega \sin \Theta \qquad (S2)$$

Where Ω is the Earths angular velocity 7.2921159×10^{-5} radians s^{-1} and θ is latitude.

Influence of Shear Environment on Storm Characteristics

(a) USA

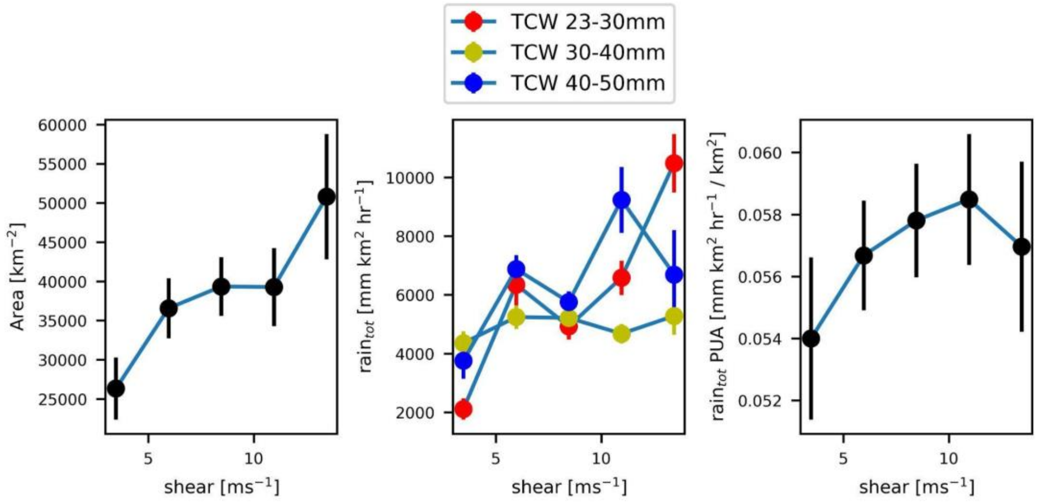

(b) Chi

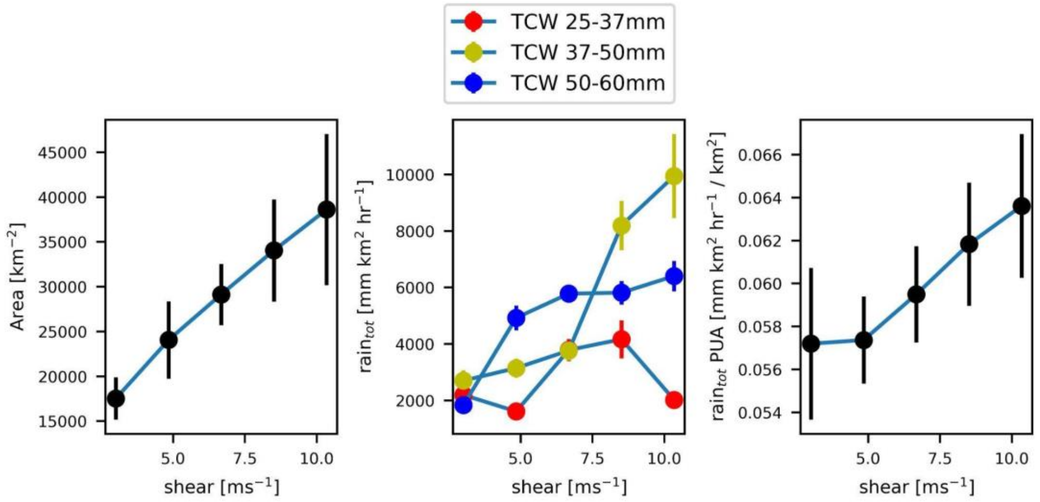

(c) Ind

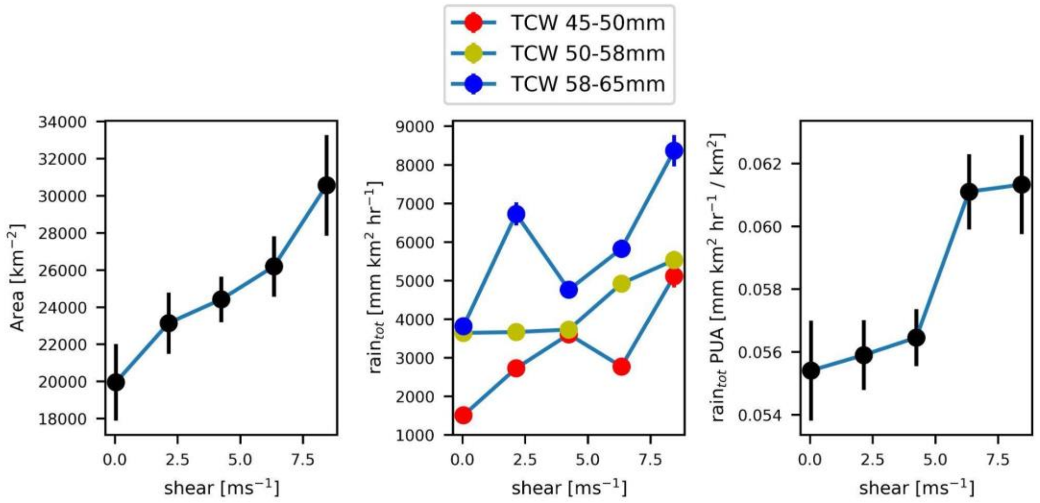

(d) WAf

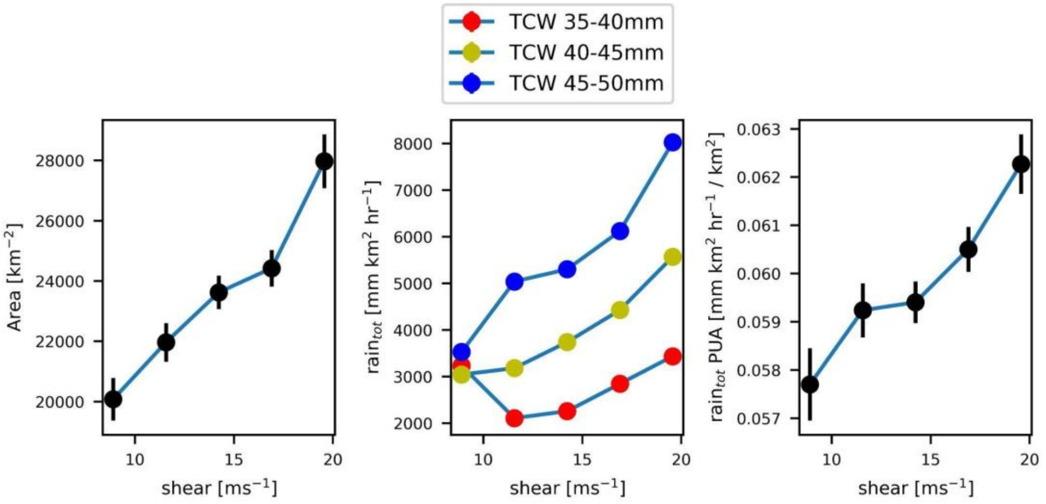

(e) Aus

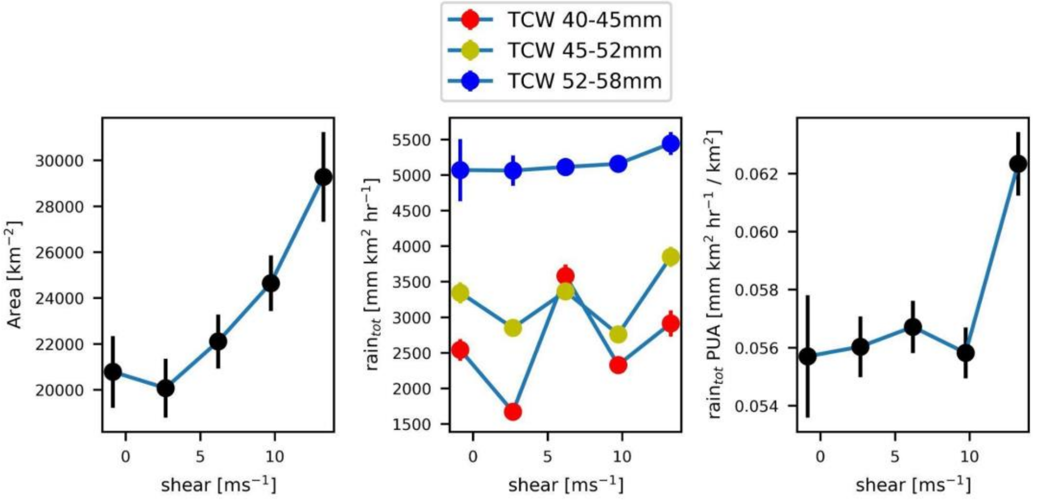

(f) SAf

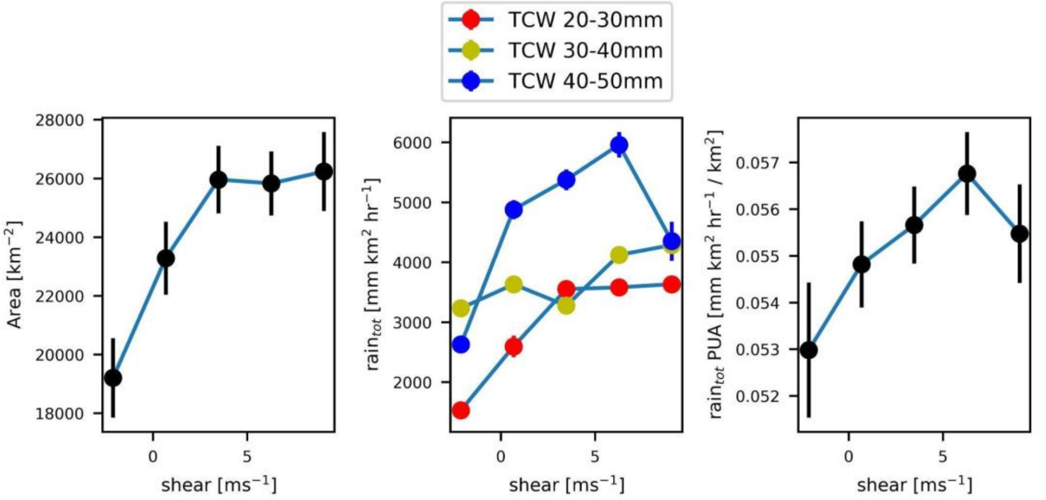

(g) SAm

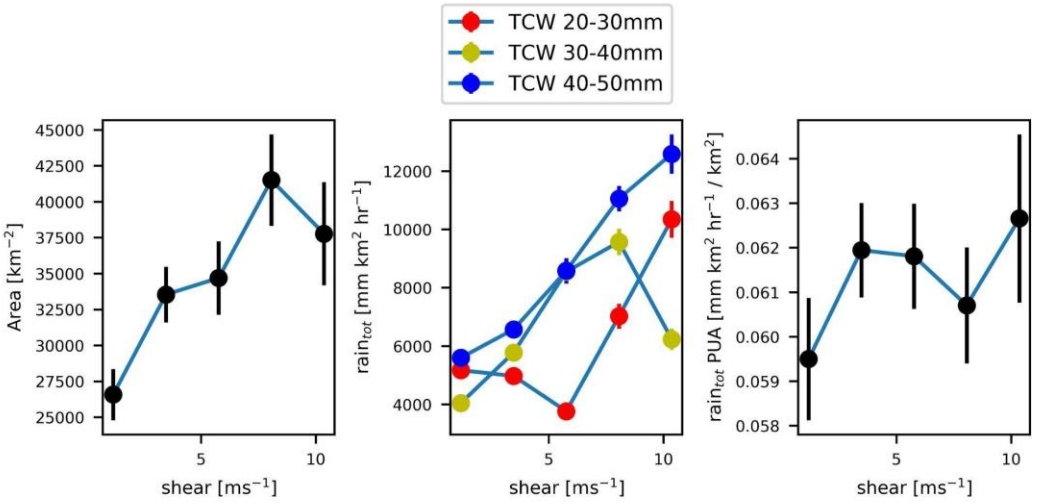

Figure S7 - Impact of shear environment on wet season storm characteristics (largest precipitation feature - PF1) for individual regions. (left) Line plot of mean PF1 area [km^2] in zonal shear bins, (middle) Line plots of 95th percentile PF1 total rainfall [mm km^2 hr^{-1}] in zonal shear bins for low (red), intermediate (yellow) and high (blue) total column water (TCW), (right) Line plot of mean total rain per unit area [mm km^2 hr^{-1} / km^2] in zonal shear bins. Data in (a) – (c) are presented as mean values + / - 95th percentile confidence intervals (n = 7 regions). Zonal shear is calculated between levels 650 hPa and 100 m. (a) Great Plains (USA), (b) China (Chi), (c) India (Ind), (d) West Africa (WAf), (e) Australia (AUS), (f) South Africa (SAf), (g) South America (SAm).

Soil moisture gradient impact on MCS characteristics

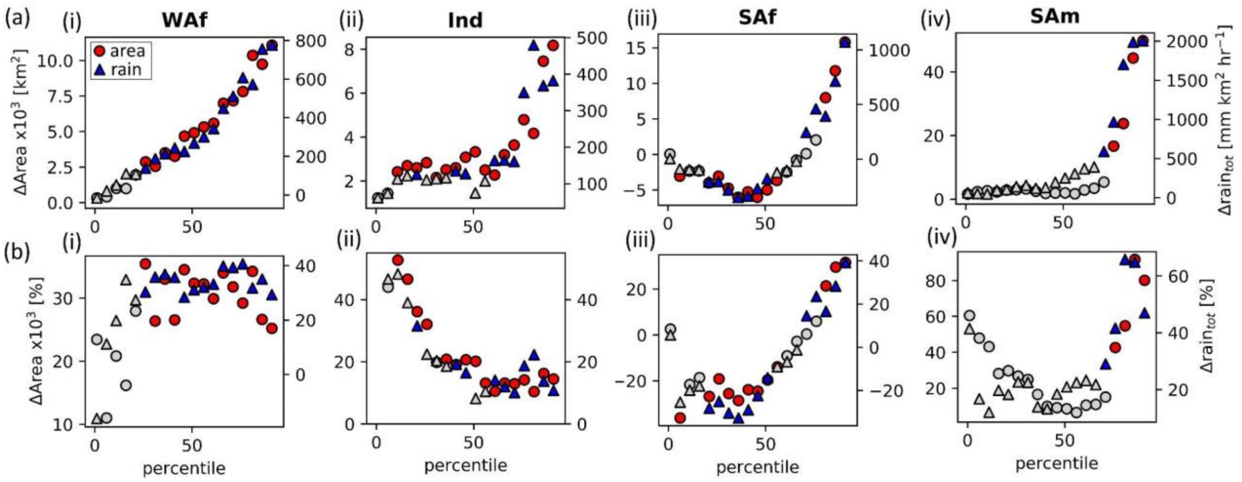

Figure S8 - Differences between wet season MCS characteristic (largest precipitation feature - PF1) distributions for favorable and unfavorable soil moisture gradient anomaly subsets in four regions. Absolute (a) and percentage (b) differences (favorable minus unfavorable) between 5th to 95th (in steps of 5) percentile values of PF1 area (circles) [km^2] and total rainfall (rain$_{tot}$, triangles) [mm km^2 hr^{-1}]. Coloured symbols represent significant ($p<0.05$) differences (i.e. differences greater than the 95th percentile confidence intervals). Favorable refers to soil moisture gradients that enhance shear in the direction of MCS propagation for that region. (i) West Africa (WAf), (ii) India (Ind), (iii) South Africa (SAf), (iv) South America (SAm).

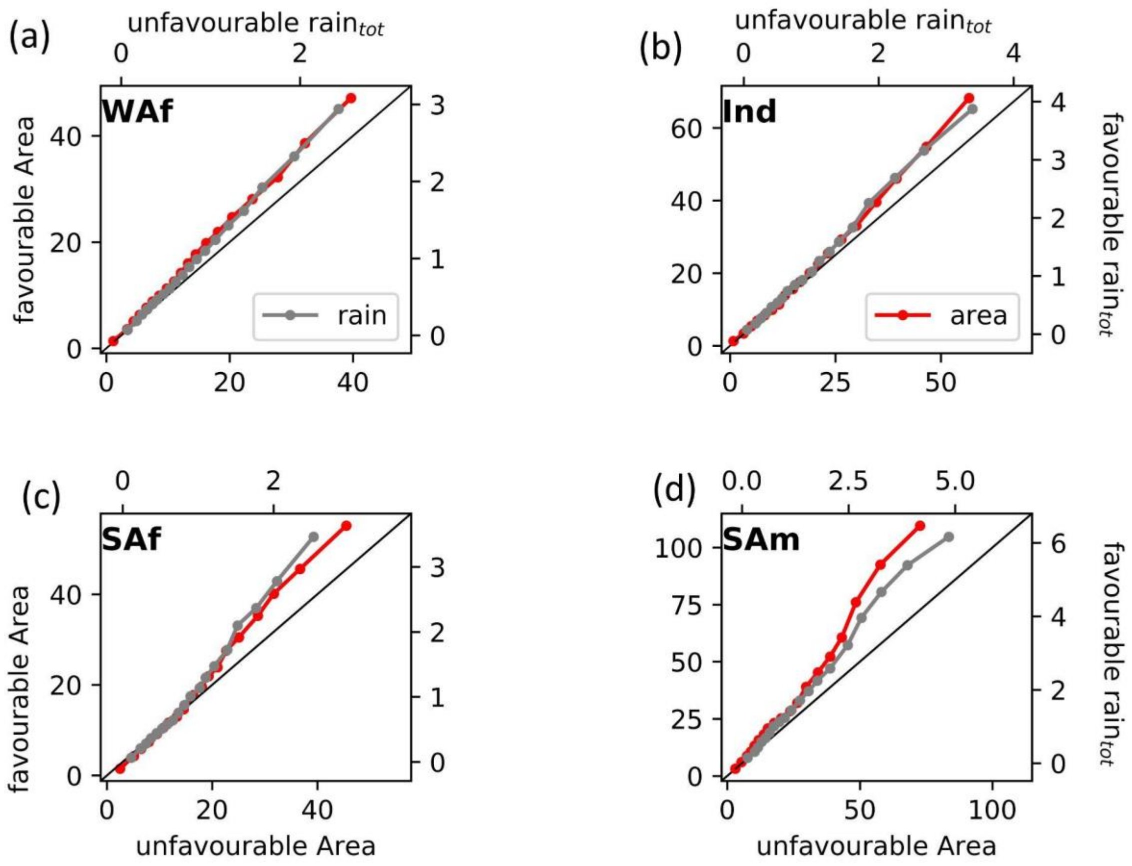

Figure S9 - Quantile-quantile (Q-Q) plots comparing 5th to 95th (in steps of 5) percentile values of PF1 area x10^3 [km^2] (red) and total rainfall [m km^2 hr^{-1}] (raintot, grey) for favourable and unfavourable soil moisture gradient anomaly (ASCAT 2015-2019) subsets. Differences in the favourable and unfavourable distributions are indicated by deviation from the 1:1 line (black). Favourable refers to soil moisture gradient anomalies that enhance shear in the direction of MCS propagation. (a) West Africa (WAf), (b) India (Ind), (c) South Africa (SAf) and (d) South America (SAm).

To evaluate whether the storm subsets analysed in Figure 3 are exposed to different thermodynamic conditions relevant to MCS characteristics, we sample the hourly evolution of domain mean (6 x 6 degree box centered on PF1 locations) convective available potential energy (CAPE) and total column water (TCW) for the period starting 120 hours before the event to 24 hours after (Figure S10).

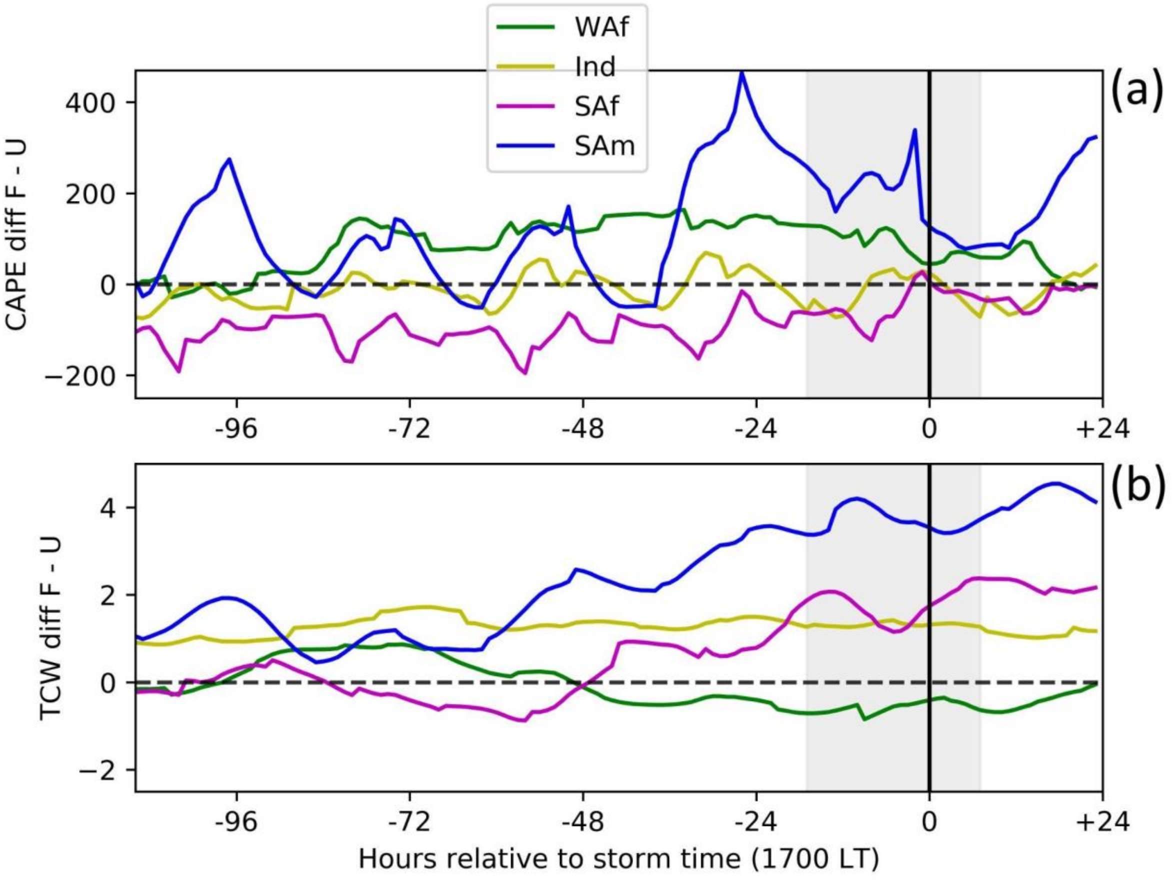

Figure S10 - Temporal evolution of differences in thermodynamic variables between soil moisture gradient anomaly (SMAP 2015-2019) subsets, favourable (F) minus unfavourable (U), in four regions. Favourable refers to soil moisture gradient anomalies that enhance shear in the direction of MCS propagation. (a) Convective available potential energy (CAPE) [J kg^{-1}] and (b) total column water (TCW) [mm].

Differences in storm day TCW (Figure S10), in particular for South America, could be contributing to observed differences in storm characteristics between soil moisture gradient anomaly subsets (Figure 3a). Therefore we perform a test where we control for storm day TCW. This is done for each region by examining the distributions of TCW for the favourable and unfavourable subsets, and then selecting a minimum and maximum TCW per region such that the mean difference between the subsets is less than 1 mm on storm day. The TCW ranges are 40-45, 48-58, 32-42 and 40-50 mm for West Africa (WAf), India (Ind), South Africa (SAf) and South America (SAm) respectively. Due to the restrictive number of cases in the SMAP subsets we create a larger sample using the ASCAT SM product (available from 2007 compared to 2015 for SMAP). Figure S11 shows the differences in thermodynamic variables between the favourable and unfavourable subsets for the controlled sample. Figure S12 shows the QQ plots for the controlled sample.

Without controlling for TCW we observe a 15-40% enhancement of the 90th percentile PF1 area and total rainfall for the favourable ASCAT subset (Figure S9). When controlling for TCW we observe a 12-30% enhancement of the 90th percentile PF1 area and total rainfall for the favourable ASCAT subset (Figure S12). The largest change is for South America, consistent with this region having the largest initial TCW differences between the subsets (Figure S10).

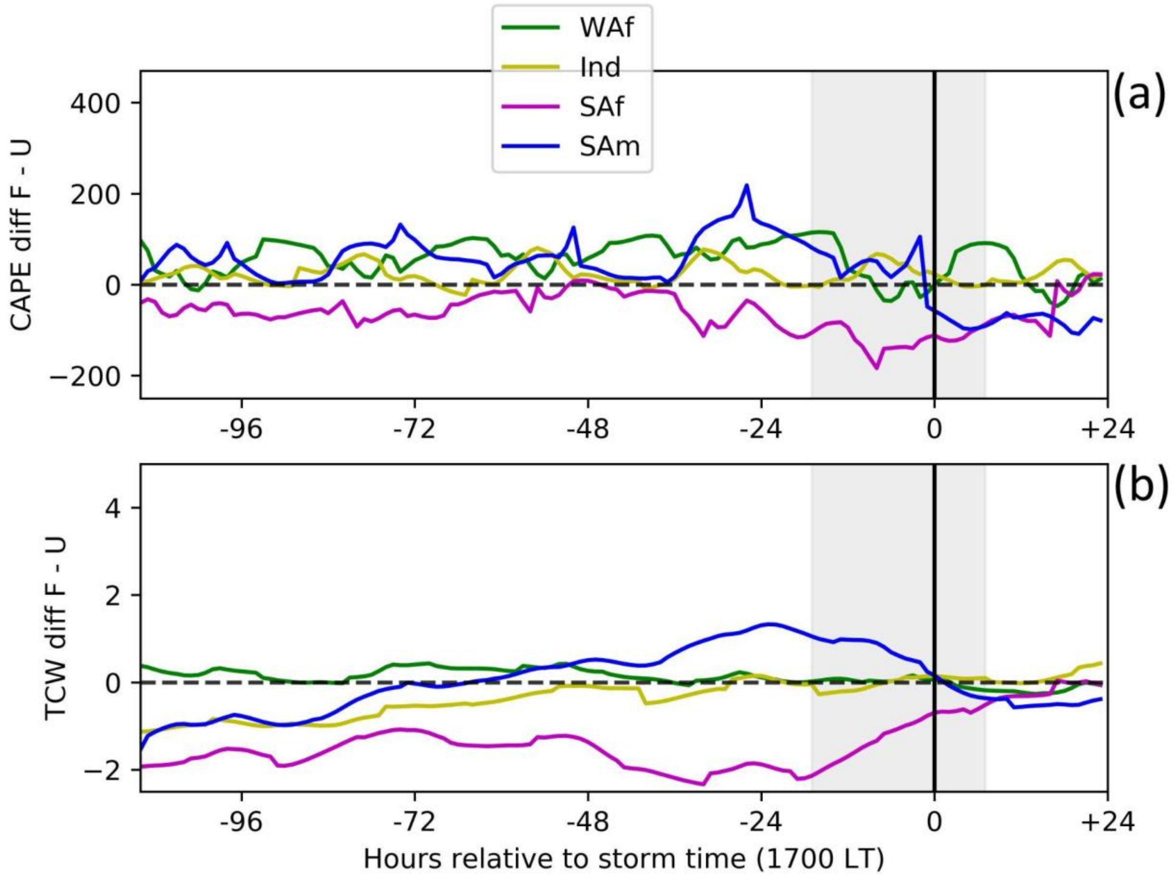

Figure S11 - Temporal evolution of differences in thermodynamic variables between soil moisture gradient anomaly (ASCAT 2007-2019) total column water (TCW) controlled subsets, favourable (F) minus unfavourable (U), in four regions. Favourable refers to soil moisture gradient anomalies that enhance shear in the direction of MCS propagation. (a) Convective available potential energy (CAPE) [J kg^{-1}] and (b) TCW [mm].

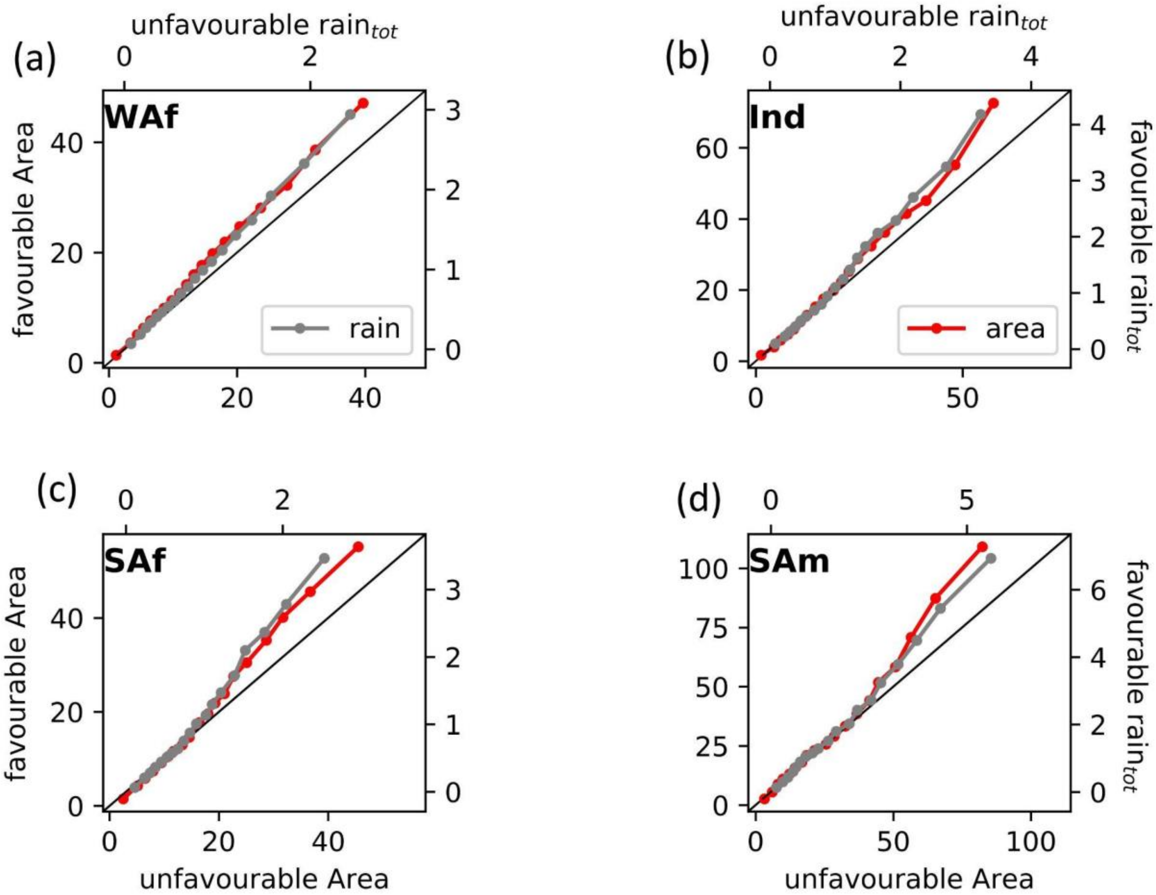

Figure S12 - Quantile-quantile (Q-Q) plots comparing 5th to 95th (in steps of 5) percentile values of PF1 area x10^3 [km^2] (red) and total rainfall [m km^2 hr^{-1}] ($rain_{tot}$, grey) for favourable and unfavourable soil moisture gradient anomaly (ASCAT 2007-2019) total column water controlled subsets. Differences in the favourable and unfavourable distributions are indicated by deviation from the 1:1 line (black). Favourable refers to soil moisture gradient anomalies that enhance shear in the direction of MCS propagation. (a) West Africa (WAf), (b) India (Ind), (c) South Africa (SAf) and (d) South America (SAm).

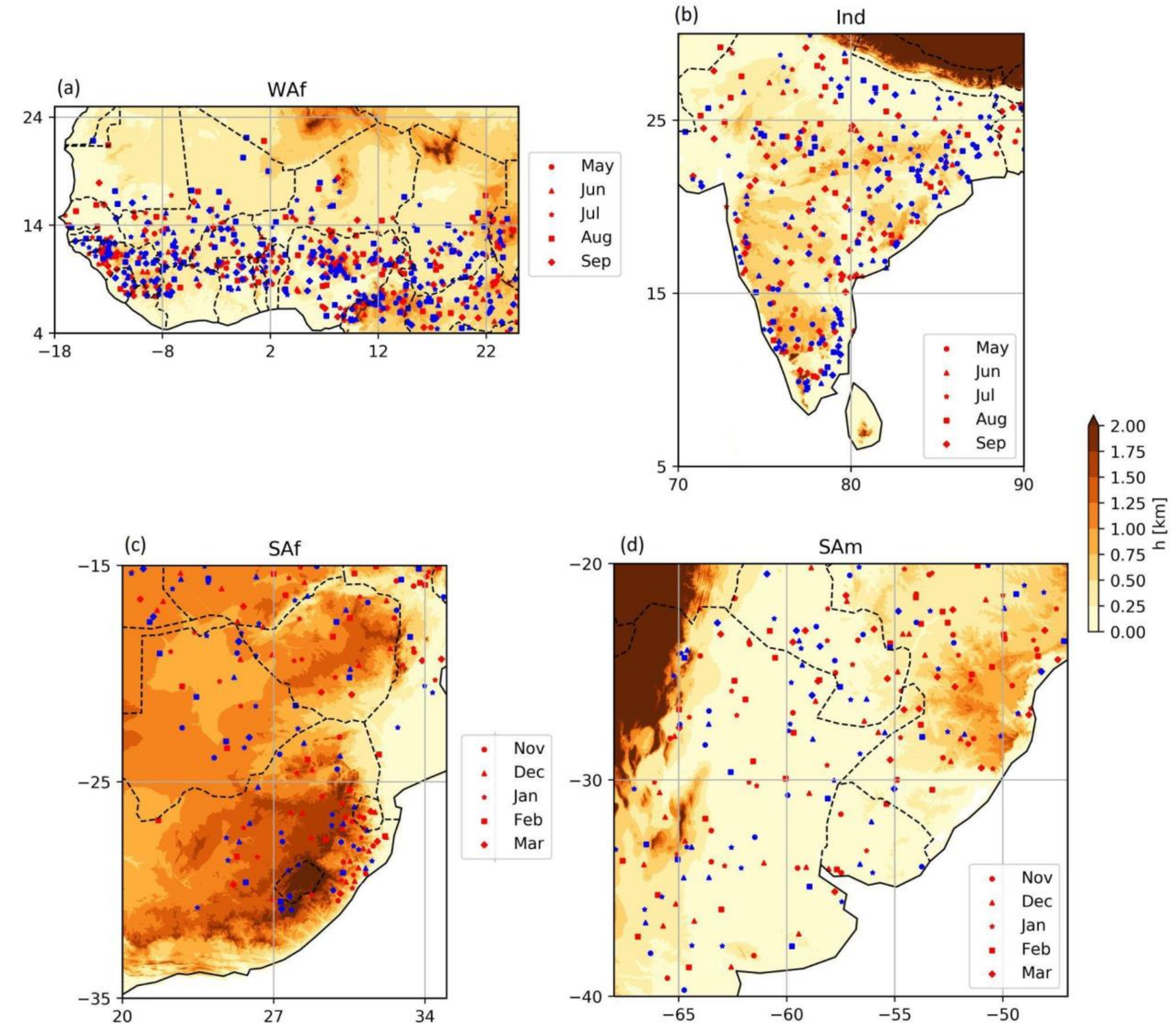

Figure S13 - Regional and temporal distribution of MCS cases in SMAP lower (blue) and upper (red) quartiles (representing the most negative and positive soil moisture gradient anomalies) overplotted on topography (h[m]). Symbols indicate month of year. (a) West Africa (WAf), (b) India (Ind), (c) South Africa (SAf), (d) South America (SAm).
